# Cross-sectional study on the current status of county-level cancer prevention and treatment capabilities in the four southwestern provinces of China

**DOI:** 10.3389/fpubh.2026.1785343

**Published:** 2026-03-27

**Authors:** Xiang Tan, Chi Du, Haibo Cai, Jinsong Xu, Yan Zhang, Yanping Li, Huade Lin, Caifan Li, Han Liang, Xudong Xiang

**Affiliations:** 1 Department of Hematology and Oncology, The People's Hospital of An'yue, Ziyang, China; 2 Oncology Department, Dujiangyan City People's Hospital, Chengdu, China; 3 Oncology Department, Xuanwei Yunfeng Hospital, Qujing, China; 4 Oncology Department, Anning City First People's Hospital, Kunming, China; 5 Oncology Department, Gejiu City People's Hospital, Honghe Prefecture, China; 6 Respiratory Medicine, Honghe Autonomous Prefecture 3rd Hospital, Honghe Prefecture, China; 7 Oncology Department, Pingnan County People's Hospital, Guigang, China; 8 Department of Thoracic Surgery, The Third Affiliated Hospital of Kunming Medical University, Yunnan Cancer Hospital, Peking University Cancer Hospital Yunnan, Kunming, China

**Keywords:** county-level cancer hospital, cross-sectional study, healthcare resources, multidisciplinary diagnosis and treatment, southwest China

## Abstract

**Background:**

The grassroots regions in the four provinces of Southwest China bear a high burden of cancer nationally. In response, the Chinese government has been continuously promoting the construction of a grassroots cancer prevention and treatment system. This study aims to systematically evaluate the current capacity for cancer prevention and treatment at the county level in this region, aligning with the “Healthy China” strategy and the “Thousand-County Project” policy objectives.

**Methods:**

This cross-sectional study was conducted from December 2023 to March 2024. A standardized online questionnaire was distributed to 470 district and county (city) level hospitals across four provinces in Southwest China (Yunnan, Sichuan, Guizhou, and Guangxi). The survey investigated the availability of hardware facilities, staffing levels, and clinical practice standards related to cancer care. Descriptive statistical analysis was used to assess the current status of cancer prevention and treatment resources and capabilities. Comparisons between groups for categorical variables were performed using the chi-square test, while continuous variables were compared using the *t*-test or Wilcoxon rank-sum test, with a significance level set at α = 0.05. All analyses were conducted using SPSS software version 22.0.

**Results:**

Tertiary hospitals dominate county-level cancer prevention and control in the four southwestern provinces (63.6%), and 46.1% of cancer prevention and control hospitals have established cancer prevention and control centers. Imaging examination coverage is high (CT/MRI coverage rates of 97.5/88.4%), and access to conventional cancer treatment methods is good (access rates for curative surgery, targeted therapy, chemotherapy, endocrine therapy, and immunotherapy are 79.1, 97.5, 96.4, 83.2% and 87.3%, respectively). Multidisciplinary diagnosis and treatment models are developing rapidly (MDT implementation rate of 56.8%), and pathology support is strong (77.8% of hospitals conduct pathological diagnosis). However, the configuration of radiotherapy equipment is significantly insufficient (0.16 linear accelerators per county on average); the rate of independently conducted gene testing is only 16.2%. The accessibility of analgesic drugs exceeds 80%.

**Conclusions:**

Basic clinical capabilities for cancer prevention and treatment at the county level in the four provinces of Southwest China are developing rapidly. The district and county (city) level cancer hospitals demonstrate high accessibility to conventional treatment modalities (surgery, chemotherapy, targeted therapy, immunotherapy, pain management, etc.) and imaging/pathological diagnostics. However, challenges remain in advanced areas of precision medicine, such as a significant lack of genetic testing capacity. Additionally, while the MDT model is progressing quickly, it still lags behind levels seen in developed countries. Finally, radiotherapy resources at the county level in this region are insufficient and unevenly distributed.

## Introduction

China bears the world's heaviest cancer burden, with incidence rising in recent years (1). In 2022, China reported approximately 4.8 million new cancer cases (24% of global total) and 2.6 million cancer deaths (26.7% of global total), both ranking first worldwide (2). Malignant neoplasms represent the leading cause of death in urban areas and the second leading in rural areas (3). Over the past two decades, China's cancer surveillance system has achieved coverage across most counties (4), highlighting challenges in grassroots cancer care—over 40% of new patients are diagnosed at this level annually (5). Rural areas demonstrate slightly lower incidence than urban areas (199.65 vs. 212.95 per 100,000) with narrowing gaps, yet face higher age-standardized 5-year mortality (103.97 vs. 92.37 per 100,000) (5, 6) and lower survival rates (33.6 vs. 46.7%) (4), indicating greater cancer burden. This disparity is particularly pronounced in Southwest China, which has the highest cancer incidence (226.7 per 100,000) and lowest survival rate (24.9%) among seven Chinese regions (7). Contributing factors include high smoking prevalence (8), relative economic underdevelopment (9), and limited public health resources (10). Additionally, variations in cancer center establishment, departmental expertise, treatment equipment availability, and standardized care may play roles—factors this study investigates further.

While the Healthy China initiative has substantially improved grassroots cancer care capacity (11, 12), challenges persist including limited treatment capabilities, insufficient medical resources, and low public awareness (13). In response, China launched the “Thousand-County Project” in 2021 to address these challenges (14). Given the unique epidemiological characteristics of Southwest China, this study investigated 470 district and county (city) level hospitals across four provinces (Yunnan, Sichuan, Guizhou, Guangxi) regarding their cancer prevention and treatment infrastructure, staffing, and clinical practices. Our findings provide evidence for optimizing grassroots cancer control policies.

## Methods

This cross-sectional study employed an onlinea questionnaire survey. Using the Wenjuanxing platform (https://www.wjx.cn/), we distributed questionnaires to oncology departments in 470 district and county (city) level hospitals across four Southwest Chinese provinces (Yunnan, Sichuan, Guizhou, Guangxi). At least one physician per hospital was randomly selected to complete the survey between December 2023 and March 2024. The questionnaire collected data on cancer prevention and treatment infrastructure, staffing, and clinical practices. All investigators received standardized training on questionnaire administration. This study, jointly initiated by 13 hospitals in Southwest China, focused exclusively on district and county (city) level hospitals, excluding provincial capitals and prefectural-level central hospitals. All participating physicians provided informed consent.

Descriptive statistics were used to characterize the current status of cancer prevention and treatment resources and capabilities in the four provinces. Categorical variables were analyzed using the chi-square test, while continuous variables were compared using the *t*-test or non-parametric Wilcoxon rank-sum test. A significance level of α = 0.05 was set, with *P* < 0.05 considered statistically significant. All analyses were performed using SPSS version 22.0.

## Results

During the study period, online questionnaires were distributed to oncology departments in all 470 district and county (city) level hospitals across four Southwest Chinese provinces. Of these, 198 hospitals had established oncology departments and returned valid questionnaires after data verification and exclusion of incomplete responses.

Based on the Seventh National Population Census data, among the 470 county- and district-level medical institutions in Sichuan, Yunnan, Guangxi, and Guizhou, 151 counties had a permanent resident population exceeding 500,000, while 319 counties had a population below 500,000. A total of 198 hospitals across 146 counties (31.1%) had established oncology departments, distributed as follows: Yunnan (68, 34.3%), Sichuan (81, 40.9%), Guangxi (28, 14.1%), and Guizhou (21, 10.6%).

**Figure 1:**
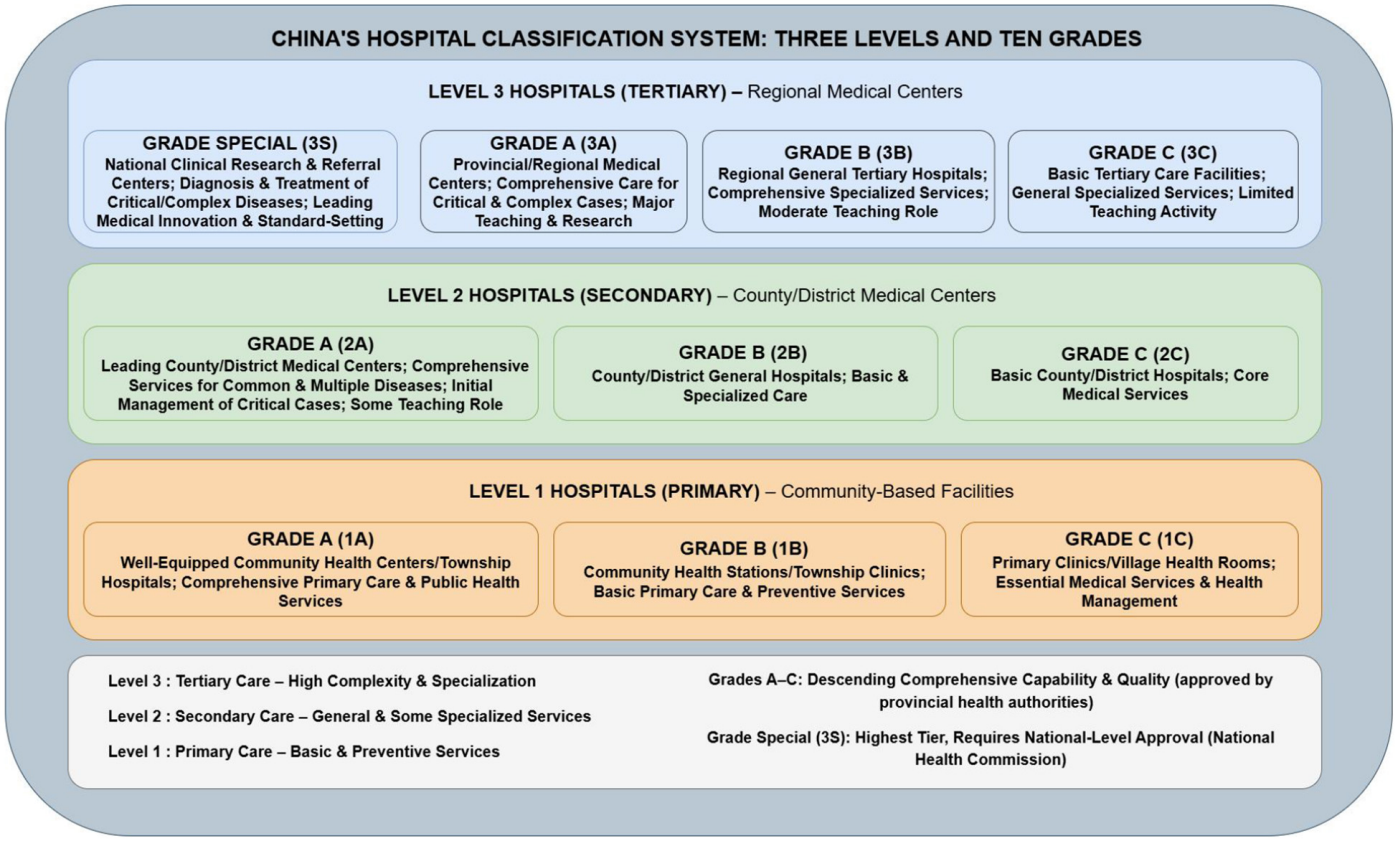
China's three-level and ten-grade hospital classification system.

Among the 198 institutions, 188 (94.9%) were public hospitals, including 186 (93.9%) general hospitals and only two (1.0%) specialized cancer hospitals. Ninety-one hospitals (46.1%) had established dedicated cancer prevention and treatment centers, with tertiary hospitals (69 centers) significantly outnumbering secondary hospitals (22 centers; *P* < 0.05; Table 1). In China's hospital grading system, hospitals are classified into three tiers (primary, secondary, tertiary) and 10 levels based on function, scale, and technical capacity, with higher tiers indicating superior medical resources and service capabilities (currently, no tertiary special-grade hospitals exist; the highest level is tertiary grade A; Figure 1). Among district and county (city) level hospitals in the four provinces, 126 (63.6%) were tertiary hospitals, including 52 (26.3%) tertiary grade A, 61 (30.8%) tertiary grade B, and 13 (6.6%) other tertiary hospitals; 72 (36.4%) were secondary hospitals, including 65 (32.8%) secondary grade A and seven (3.5%) secondary grade B (Table 1). Thus, cancer prevention and treatment at the county level in Southwest China primarily rely on oncology departments within public tertiary general hospitals.

**Table 1:** Current status of regional tumor control institutions.

Research indicator	Category	*n*	Percentage (%)	Cumulative percentage (%)
Distribution of tumor control hospitals	Sichuan	81	40.9	40.9
	Yunnan	68	34.3	75.3
	Guizhou	21	10.6	85.9
	Guangxi	28	14.1	100
Type of hospitals	Public general hospitals	186	93.9	93.9
	Public specialized tumor hospitals	2	1.0	94.9
	Private general hospitals	8	4.1	99
	Private specialized tumor hospitals	2	1.0	100
Hospital level	Tertiary grade A	52	26.3	26.3
	Tertiary grade B	61	30.8	57.1
	Other tertiary hospitals	13	6.6	63.6
	Secondary grade A	65	32.8	96.5
	Secondary grade	7	3.5	100
Cancer prevention and treatment center	Yes	91	46	46
	No	107	54	100

**Figure 2:**
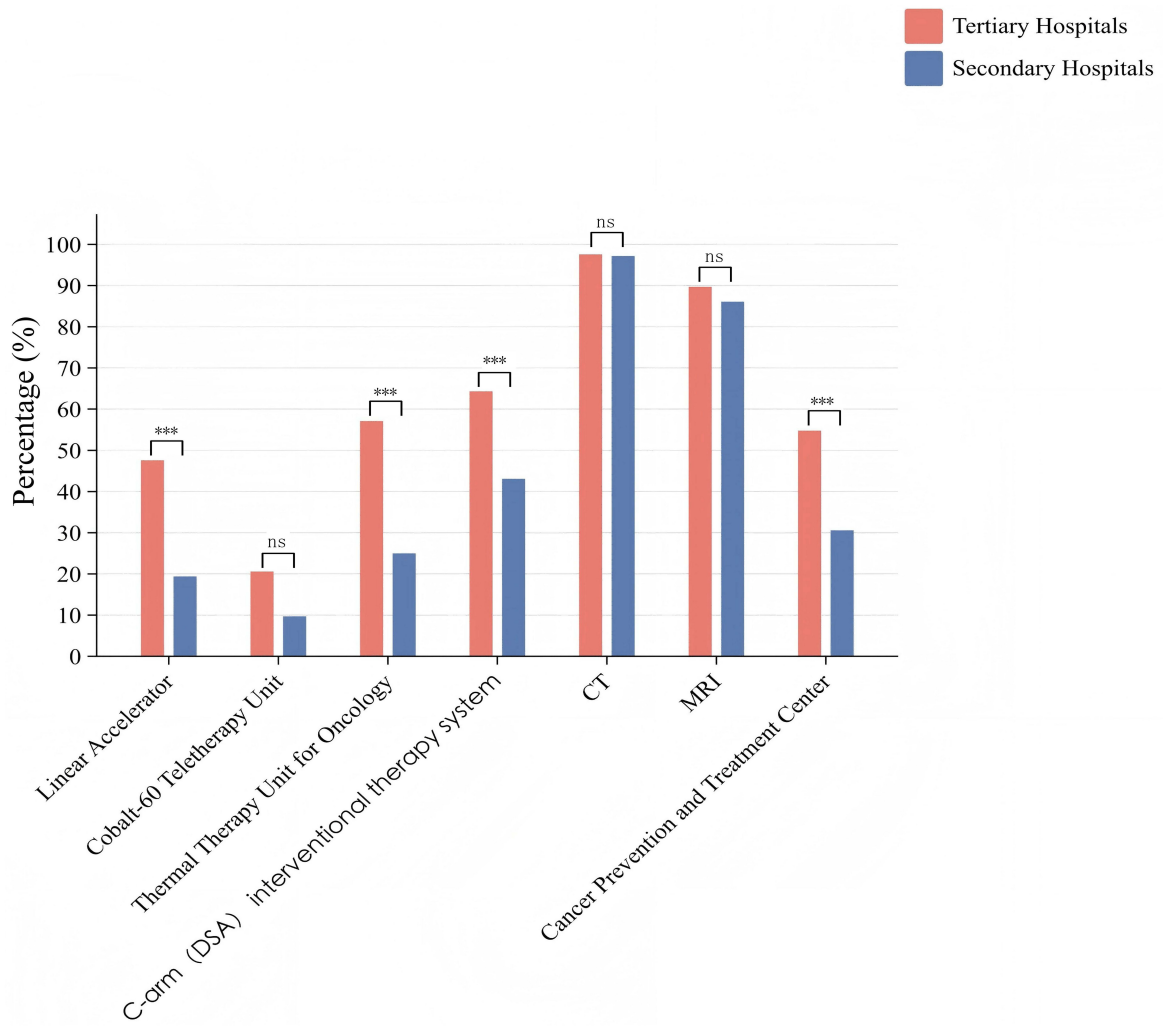
Current status of hardware equipment configuration for malignant tumor diagnosis and treatment at the county level in the four provinces of Southwest China. *P* ≥ 0.05; *P < 0.05; **P < 0.01; ***P < 0.001.

Among the 198 district and county (city) level hospitals surveyed, 74 (37.4%) were equipped with linear accelerators, 33 (16.7%) with cobalt-60 therapy units, 90 (45.5%) with hyperthermia machines, and 112 (56.6%) with C-arm DSA interventional systems. Further analysis revealed that tertiary hospitals had significantly higher equipment rates than secondary hospitals for linear accelerators, hyperthermia machines, and C-arm DSA systems (all *P* < 0.001; Supplementary Table S1, Figure 2). Sichuan Province, with its relatively developed economy, demonstrated significant advantages over other provinces in both the proportion of tertiary hospitals and the availability of linear accelerators, cobalt-60 units, hyperthermia machines, and C-arm DSA systems (*P* < 0.001; Supplementary Table S2).

**Figure 3:**
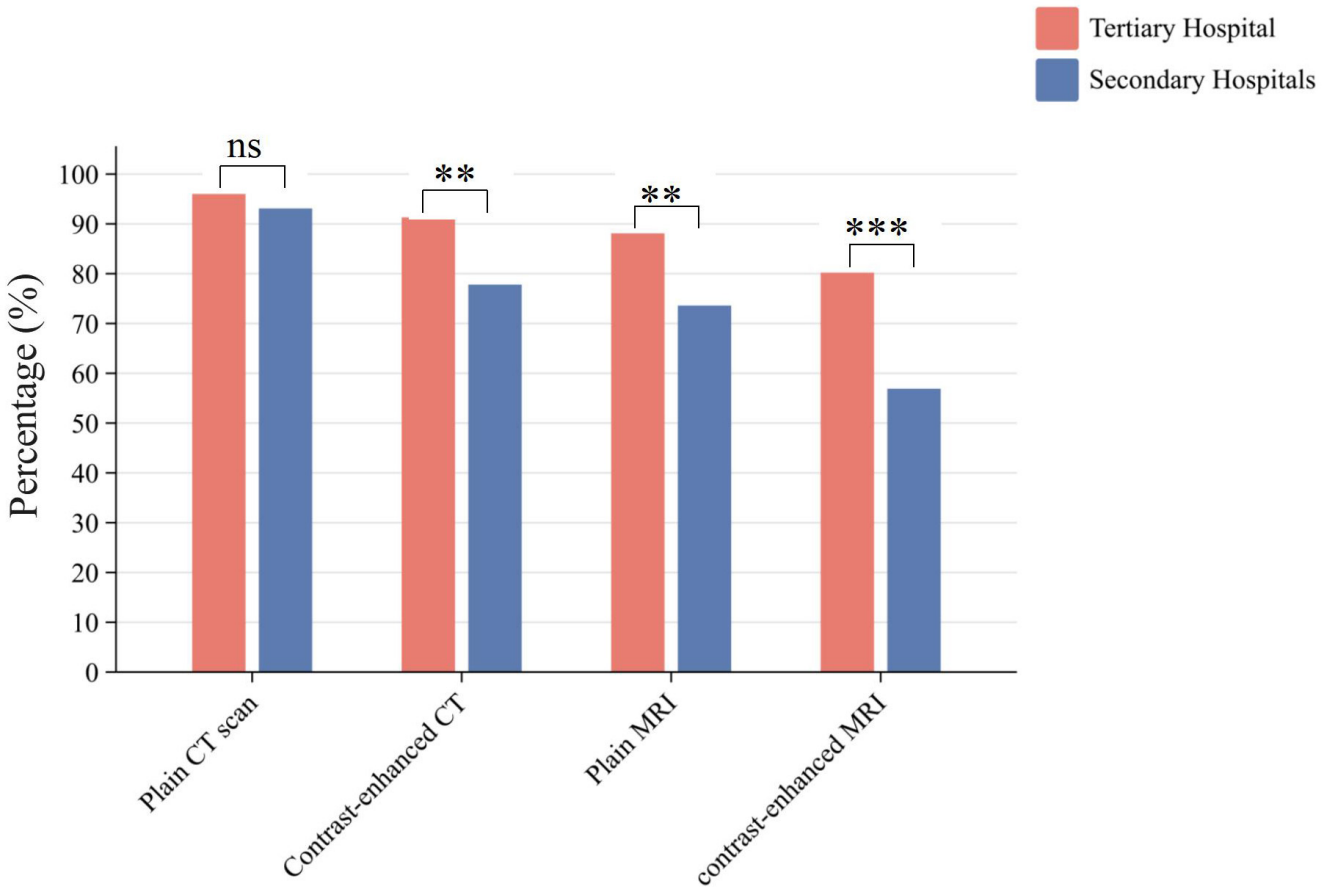
Implementation status of imaging department services at the county level in the four provinces of Southwest China. *P* ≥ 0.05; *P < 0.05; **P < 0.01; ***P < 0.001.

Regarding imaging capabilities, 193 hospitals (97.5%) were equipped with computed tomography (CT) systems, including 97.6% (*n* = 123) of tertiary hospitals and 97.2% (*n* = 70) of secondary hospitals. Additionally, 175 hospitals (88.4%) were equipped with magnetic resonance imaging (MRI) systems, including 89.7% (*n* = 113) of tertiary hospitals and 86.1% (*n* = 62) of secondary hospitals (Supplementary Tables S1, S3). Utilization rates for imaging modalities were as follows: plain CT 94.9%, contrast-enhanced CT 91.3%, plain MRI 82.8%, and contrast-enhanced MRI 71.7%. CT and MRI equipment rates were uniformly high across provinces and hospital levels, with no significant differences observed (*P* > 0.05). However, tertiary hospitals demonstrated significantly higher contrast-enhanced MRI utilization rates compared with secondary hospitals (*P* < 0.001; Figures 2, 3; Supplementary Tables S1, S3).

**Figure 4:**
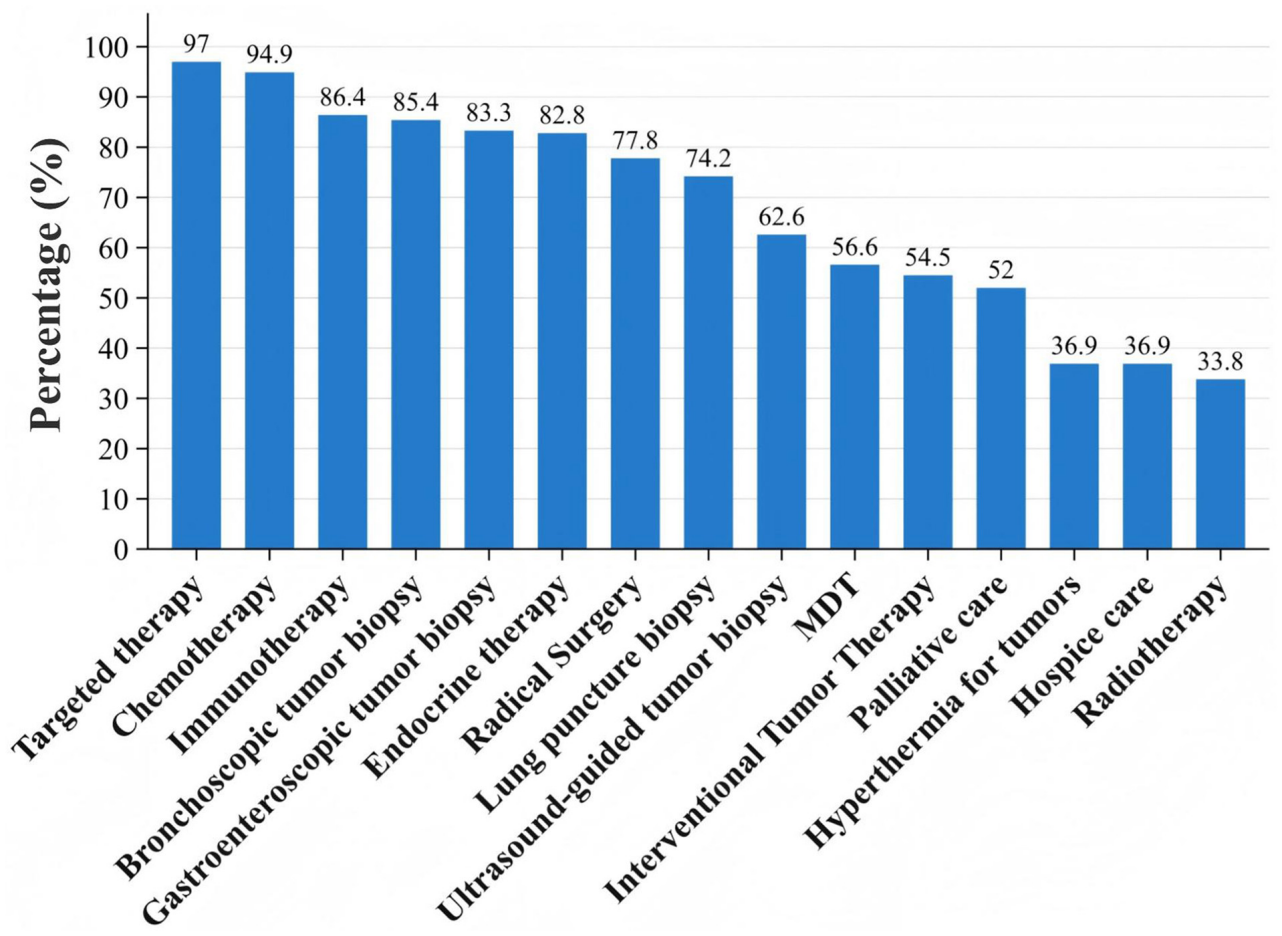
Current status of professional technical services in the field of oncology at county-level in the four southwestern provinces.

In terms of clinical oncology service availability, the rates were: curative surgery 79.1%, chemotherapy 96.4%, endocrine therapy 83.2%, targeted therapy 97.5%, immunotherapy 87.3%, interventional therapy 54.8%, radiotherapy 33.8%, and palliative care 37.0%. Chemotherapy, endocrine therapy, and targeted therapy showed high availability across all hospital levels without significant differences (*P* > 0.05). Tertiary hospitals outperformed secondary hospitals in curative surgery and immunotherapy (*P* < 0.05). Radiotherapy availability was low across all hospital levels, with the majority of resources (83.6%) concentrated in large tertiary hospitals (*P* < 0.001). Multidisciplinary team (MDT) discussions for complex cases were conducted in 56.8% of hospitals, with significantly higher rates in tertiary hospitals compared with secondary hospitals (69.8% vs. 47.2%, *P* < 0.001). Endoscopic, ultrasound-guided, and CT-guided tumor biopsy techniques were widely available (Table 2, Figure 4).

**Table 2:** Current status of oncology specialty technical services at hospitals of different levels.

Service item	All hospitals [*n* (%)]	Tertiary hospitals [*n* (%)]	Secondary hospitals [*n* (%)]	χ^2^	*P*
Chemotherapy	188 (94.9)	121 (96)	67 (93.0)	0.846	0.358
Endocrine therapy	164 (82.8)	109 (86.5)	55 (76.3)	3.299	0.69
Targeted therapy	192 (97.0)	123 (97.6)	68 (94.4)	0.583	0.445
Immunotherapy	171 (86.4)	116 (92.0)	55 (76.3)	9.559	0.002
Radical Surgery	154 (77.8)	111 (88.0)	43 (59.7)	21.341	< 0.001
Radiotherapy	67 (33.8)	56 (44.4)	11 (15.2)	17.410	< 0.001
Interventional Tumor Therapy	108 (54.5)	77 (61.1)	31 (43.0)	6.475	0.011
Hyperthermia for tumors	73 (36.9)	59 (46.8)	14 (19.4)	14.758	< 0.001
Palliative care	103 (52)	75 (59.5)	28 (38.8)	7.817	0.005
MDT	112 (56.6)	83 (65.8)	29 (40.2)	13.472	< 0.001
Hospice care	73 (36.9)	58 (46.0)	15 (20.8)	12.449	< 0.001
Lung puncture biopsy	147 (74.2)	106 (84.1)	41 (56.9)	17.704	< 0.001
Ultrasound-guided tumor biopsy	24 (62.6)	91 (72.2)	33 (45.8)	13.632	< 0.001
Bronchoscopic tumor biopsy	169 (85.4)	116 (92.1)	53 (73.6)	12.479	< 0.001
Gastroenteroscopic tumor biopsy	165 (83.3)	115 (91.3)	50 (69.4)	15.714	< 0.001

In terms of pathological diagnosis, 166 hospitals (83.8%) had established pathology departments, including 90.5% (*n* = 114) of tertiary hospitals and 72.2% (*n* = 52) of secondary hospitals. Among these, 154 hospitals (77.8%) could routinely perform pathological diagnosis, including 90.5% (*n* = 114) of tertiary hospitals and 55.6% (*n* = 40) of secondary hospitals, providing strong support for precision cancer prevention and treatment at the county level. Notably, only 32 hospitals (16.2%) could independently perform in-house genetic testing, while the remaining 166 hospitals (83.8%) referred genetic testing to third-party laboratories or higher-level hospitals. Further analysis revealed that tertiary hospitals had significantly superior pathological diagnostic capabilities compared with secondary hospitals (*P* < 0.001); however, access to genetic testing was low across both hospital levels (Table 3).

**Table 3:** Current status of pathology development at hospitals of different levels.

Service item	All hospitals [*n* (%)]	Tertiary hospitals [*n* (%)]	Secondary hospitals [*n* (%)]	χ^2^	*P*
Possession of a pathology department	166 (83.8)	114 (90.5)	52 (72.2)	11.267	< 0.001
Routine pathological diagnosis	154 (77.8)	114 (90.5)	40 (55.6)	30.327	< 0.001
Genetic testing	32 (16.2)	27 (21.4)	5 (6.9)	7.094	0.008

Regarding analgesic medications, which play a crucial role in pain management and quality of life for cancer patients, non-steroidal anti-inflammatory drugs (NSAIDs) showed the highest availability at 88.3%. Overall, access to analgesic medications exceeded 80%.

**Figure 5:**
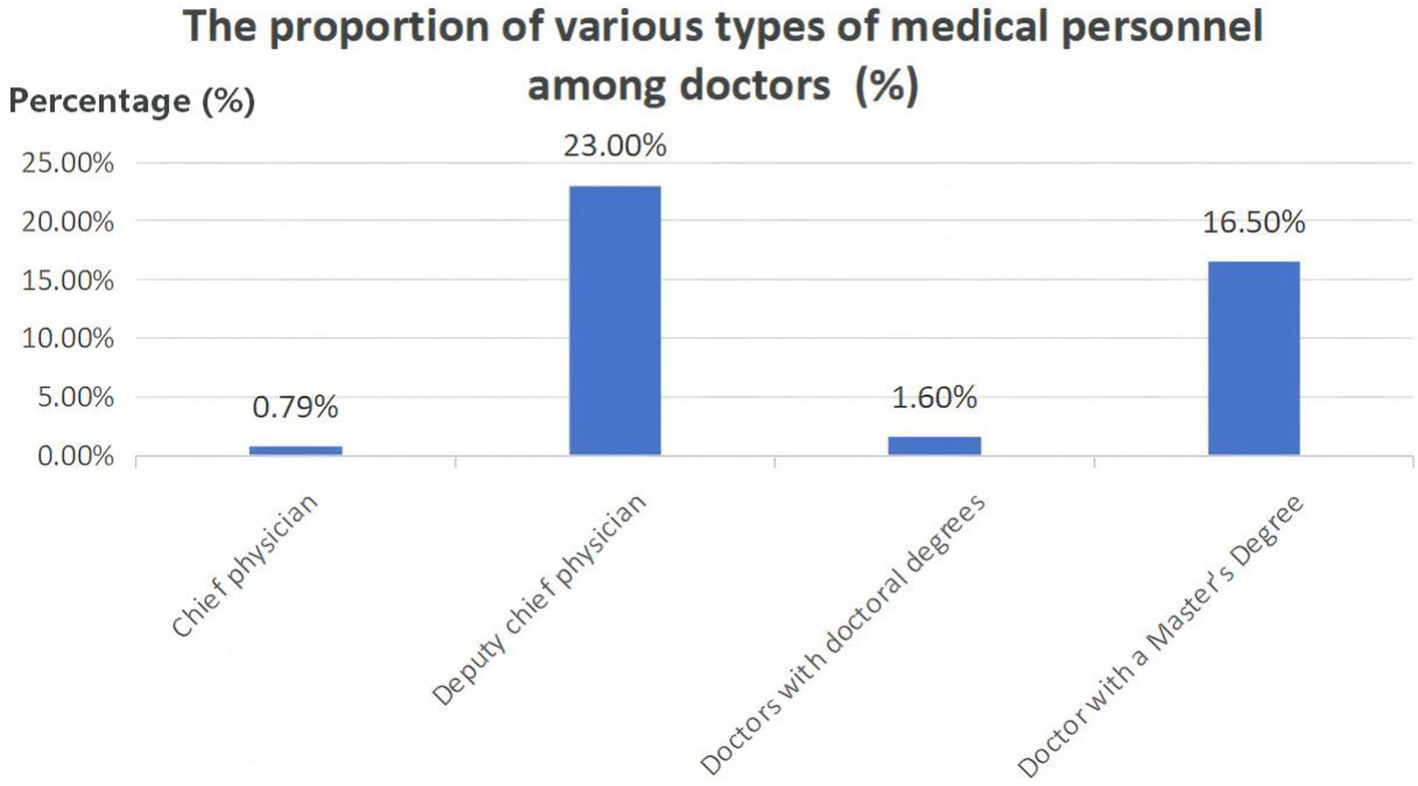
Current status of medical personnel equipped for cancer prevention and treatment at the county-level in the four provinces of southwestern China.

We also investigated staffing levels in oncology departments across the 198 district and county (city) level hospitals. The district and county (city) level oncology departments in the four Southwest provinces had an average of 8.03 ± 7.47 physicians and 14.90 ± 11.81 nurses per hospital (Supplementary Table S4). Among all physicians, approximately 30.90% held senior professional titles, and 17.10% possessed a master's degree or higher; however, the average number of chief physicians and doctoral degree holders per hospital was less than one (Figure 5). Additionally, the average number of radiation physicists per hospital was only 0.74 ± 1.27, and radiation therapists averaged 0.74 ± 1.25 (Supplementary Table S4).

## Discussion

This study provides the first systematic evaluation of cancer prevention and treatment capabilities in grassroots district and county (city) level hospitals across four Southwest Chinese provinces, focusing on institutions responsible for primary healthcare services. Our findings demonstrate rapid development in standardized cancer diagnosis, treatment, and nursing care within the regional grassroots system. However, significant gaps remain in advanced technologies, particularly the shortage and uneven distribution of radiotherapy resources. Cancer care delivery relies pre-dominantly on public tertiary hospitals (63.6%), a pattern consistent with China's tiered healthcare system, where provincial- and municipal-level large general hospitals command superior medical resources and advanced technologies, playing pivotal roles in elevating overall oncology care standards and efficiency (15, 16). Given that cancer management typically requires sophisticated equipment (e.g., CT, MRI, linear accelerators) and multidisciplinary expertise, oncology services are primarily delivered by secondary and tertiary hospitals. Consequently, our study exclusively included hospitals with established cancer care capabilities. Tertiary hospitals demonstrated significant advantages over secondary institutions across pathology, surgery, radiotherapy, multidisciplinary team (MDT) implementation, and genetic testing, establishing their dominant position in regional cancer care.

Regarding basic diagnostic and treatment capabilities, district and county (city) level hospitals in Southwest China exhibited strong accessibility. First, basic imaging examinations were highly accessible. Effective early diagnosis is crucial for reducing cancer mortality (17), with imaging serving as the cornerstone for early screening and precision diagnosis (18). Li et al. reported that by late 2022, CT coverage in Chinese secondary and tertiary hospitals reached 96.6% (19). The 2023 National Health Commission's “Equipment Allocation Standards for County-Level General Hospitals” mandates at least one CT and one MRI per county hospital, with adjustments based on hospital scale (20). Our study found high CT (97.5%) and MRI (88.4%) coverage in Southwest county hospitals, with minimal disparity between tertiary and secondary institutions, meeting national standards and aligning with national averages. Furthermore, conventional treatment modalities were widely accessible: curative surgery (79.1%), chemotherapy (96.4%), targeted therapy (97.5%), and cancer pain management capabilities satisfied most patients' basic needs. These foundational capacities provide essential support for county-level cancer care in Southwest China.

Additionally, pathology services have developed rapidly in the region. Pathological diagnosis is critical for precision oncology (21). A 2016–2019 survey by the Chinese Medical Association across 25 provinces revealed that Southwest China accounted for approximately 17.6% of national pathology departments, lagging behind eastern coastal regions (22). Currently, our study shows that 83.8% of hospitals in the region have established pathology departments, with 77.8% capable of conducting standardized pathological diagnoses.

However, it should be noted that the capacity for pathological diagnosis in county-level hospitals within the region (77.8%) still lags behind the capacity for systematic cancer treatment (chemotherapy, targeted therapy, and immunotherapy availability all exceeding 85%). This phenomenon of “treatment capability outpacing diagnostic capability” is largely attributable to China's recent vigorous promotion of integrated medical alliances and the development of independent clinical laboratories. Firstly, with the advancement of compact county-level medical communities, many regions in China have established regional pathological diagnosis centers (23). These district and county (city) level hospitals can access homogenized pathological diagnostic services through a model of “sample collection at grassroots level, central diagnosis, and remote reporting.” Secondly, some district and county (city) level hospitals meet the demand for precision medication by outsourcing pathological testing to third-party laboratories through government procurement of services.

Nevertheless, district and county (city) level hospitals face significant challenges in the advanced domains of precision cancer care. Firstly, radiotherapy capacity is markedly insufficient. The World Health Organization estimates that 50%-70% of cancer patients require radiotherapy (24, 25). However, our data reveal that the county-level radiotherapy availability in this region is only 33.8%, with the allocation rate of linear accelerators, a critical radiotherapy device, standing at just 37.4%. Although this represents an improvement compared to the 2019 availability rate in Western China (23.4%) (26), it still lags considerably behind the Eastern region (48.9% in 2019) and developed countries (e.g., overall radiotherapy availability >70% in the United States) (27). Overall, the average number of linear accelerators per county in the four Southwest provinces is only 0.16, with some counties in Yunnan and Guizhou having less than one unit per million population. Consequently, the overall allocation of radiotherapy resources at the county level in Southwest China is inadequate, and these resources are pre-dominantly concentrated in large tertiary hospitals. This inequitable distribution may lead to treatment interruptions or force patients to seek care outside their local areas.

Furthermore, our data show that while 97.5% of county-level cancer hospitals in the four Southwest provinces can provide targeted therapy, only 16.2% can independently perform in-house genetic testing. Constrained by high costs and the technical complexity of next-generation sequencing (NGS), genetic testing (including ctDNA analysis, etc.) (28) is rarely conducted independently within county hospitals. A substantial 83.8% of district and county (city) level institutions must outsource these tests to third-party laboratories or higher-level medical centers with the requisite capabilities. The lack of genetic testing capacity in Southwest China will increase the cost of targeted therapy for grassroots cancer patients and compromises the standardization of targeted drug use (29). Finally, the Multi-Disciplinary Treatment (MDT) model, while developing rapidly, still lags behind developed countries. A survey by Yang Linghe et al. found that in 2019, the median MDT implementation rate in provincial tertiary hospitals in China was only 3.98% (30). Our study reveals that currently, 56.8% of county-level cancer prevention and treatment hospitals in the four Southwest provinces have adopted the MDT model for complex cases. In contrast, over 65% of medical institutions in Europe and the United States have implemented the oncology MDT model; for instance, by 2004, more than 80% of cancer patients in the UK were managed through MDT (31). These data indicate that while the MDT model is undergoing rapid development in district and county (city) level medical institutions in Southwest China, a gap persists between China and Western countries overall. There remains a lack of standardized guidelines to guide the high-quality development of the MDT model.

In China's cancer prevention and control system, provincial and pre-fectural-level central hospitals are responsible for managing complex and severe cases and leading the advancement of sophisticated technologies, whereas county-level hospitals are tasked with screening for common cancers, providing routine diagnosis and treatment, managing rehabilitation, and facilitating bidirectional referrals. This establishes a division of labor wherein provincial-level institutions provide advanced technological support, and county-level institutions deliver foundational treatment and rehabilitation. A recent study by Wang et al. indicated that 82.9% of county-level cancer hospitals have established collaborative partnerships with higher-level hospitals, with a referral rate of 79.4% between different tiers of institutions (11). Regarding government funding allocation, national and provincial regional medical centers located in provincial capitals and major cities are primarily funded by governments at or above the provincial level, concentrating the most high-quality medical resources. In contrast, county-level hospitals rely mainly on local fiscal allocations and their own revenue generation, creating an inherent disadvantage in acquiring advanced technologies. However, in recent years, the national government has explicitly increased investment in county-level hospitals, enhancing funding, implementing personnel subsidies, and providing operational support for underdeveloped departments (14).

This study provides a comprehensive investigation of data from district and county (city) level hospitals across four provinces in Southwest China, offering an integrated assessment of the current state of cancer prevention and treatment in grassroots hospitals within this region. It reflects both the strengths and deficiencies of grassroots cancer care in this area. Nevertheless, this study surveyed only district and county (city) level hospitals in these four southwestern provinces; therefore, the findings may not be fully representative of the national landscape of grassroots cancer prevention and treatment. Furthermore, as a cross-sectional study, it does not allow for dynamic observation of changes in the cancer care capabilities of district and county (city) level hospitals over time.

## Conclusion

This study demonstrates rapid development of foundational cancer care capabilities in county-level hospitals across four Southwest Chinese provinces. Led by tertiary hospitals, grassroots institutions exhibit high accessibility to conventional treatment modalities (surgery, chemotherapy, targeted therapy, immunotherapy, pain management) and imaging/pathological diagnostics, essentially meeting routine care needs of regional cancer patients. However, district and county (city) level hospitals continue to face challenges in advanced precision medicine domains, including limited genetic testing capacity and a multidisciplinary team (MDT) model that, despite rapid progress, still lags behind developed countries. Furthermore, radiotherapy resources at the county level remain insufficient and unevenly distributed.

Future policy priorities should therefore accelerate the development of cancer prevention and treatment centers, leveraging the technical outreach of large tertiary hospitals to expedite the adoption of advanced technologies such as genetic testing in lower-tier hospitals, while standardizing and promoting the widespread implementation of the MDT model. Concurrently, efforts to allocate radiotherapy resources in county-level hospitals must be intensified.

## Author's note

Preliminary data from this study were accepted as a poster presentation at the ASCO Breakthrough meeting and published as an abstract in the JCO supplement.

## Data Availability

The raw data supporting the conclusions of this article will be made available by the authors, without undue reservation.
