# Supplementary material for: Cross-sectional study on the current status of county-level cancer prevention and treatment capabilities in the four southwestern provinces of China
Source: Front Public Health. 2026 Mar 27;14:1785343. doi: 10.3389/fpubh.2026.1785343 (PMC13066248; doi:10.3389/fpubh.2026.1785343)
Supplement: Supplementary file 1 [file Table_1.docx]

Supplementary Table S1 Current Status of Diagnostic and Therapeutic Hardware Equipment for Malignant Tumors at Different Levels

|  | All hospitals（n（%）） | Tertiary hospitals（n（%）） | Secondaryhospitals（n（%）） | χ2 | P |
| --- | --- | --- | --- | --- | --- |
| Linear Accelerator | 74(37.4) | 60（47.6） | 14(19.4) | 15.539 | <0.001 |
| Cobalt-60 Teletherapy Unit | 33(16.7) | 26（20.6） | 7(9.7) | 2.507 | 0.113 |
| Thermal Therapy Unit for Oncology | 90(45.5) | 72（57.1） | 18(25.0) | 19.093 | <0.001 |
| C-arm（DSA） interventional therapy system | 112(56.6) | 81（64.3） | 31(43.1) | 28.286 | <0.001 |
| CT | 193(97.5) | 123（97.6） | 70(97.2) | 0.029 | 0.864 |
| MRI | 175(88.4) | 113(89.7) | 62(86.1) | 0.569 | 0.451 |
| Cancer Prevention and Treatment Center | 91（46.1） | 69（54.8） | 22（30.6） | 10.809 | 0.001 |

Supplementary Table S2 Distribution of Hardware Equipment for Malignant Tumor Diagnosis and Treatment Across Provinces

|  | All hospitals  （n（%）） | Sichuan （n（%）） | Yunnan  （n（%）） | Guangxi（n（%）） | Guizhou  （n（%）） | χ2 | P |
| --- | --- | --- | --- | --- | --- | --- | --- |
| Linear Accelerator | 74(37.4) | 36（44.4） | 14（20.6） | 18（85.7） | 6（21.4） | 33.924 | <0.001 |
| Cobalt-60 Teletherapy Unit | 33(16.7) | 12（14.8） | 6（8.8） | 12（57.1） | 3（10.7） | - | <0.001 |
| Thermal Therapy Unit for Oncology | 90(45.5) | 46（56.8） | 16（23.5） | 16（76.2） | 12（42.9） | 25.460 | <0.001 |
| C-arm（DSA） interventional therapy system | 112(56.6) | 47（58.0） | 31（45.6） | 14（66.7） | 20（71.4） | 6.795 | 0.078 |
| CT | 193(97.5) | 81（100.0） | 65（95.6） | 19（90.5） | 28（100.0） | - | 0.032 |
| MRI | 175(88.4) | 71（87.7） | 60（88.2） | 19（90.5） | 24（85.7） | - | 0.963 |
| Numbers of tertiary hospitals | 126（63.6） | 72（88.9） | 24（35.2） | 19（90.5） | 11（39.3） | 59.639 | <0.001 |

Supplementary Table S3 Technical Services Offered by Radiology Departments at Hospitals of Different Levels

|  | All hospitals（n（%）） | Tertiary hospitals（n（%）） | Secondary hospitals（n（%）） | χ2 | P |
| --- | --- | --- | --- | --- | --- |
| Plain CT scan | 188（94.9） | 121（96.0） | 67（93.1） | 0.846 | 0.358 |
| Contrast-enhanced CT | 171（86.3） | 115（91.3） | 56（77.8） | 7.082 | 0.008 |
| Plain MRI scan | 164（82.8） | 111（88.1） | 53（73.6） | 6.758 | 0.009 |
| Contrast-enhanced MRI | 142（71.7） | 101（80.2） | 41（56.9） | 12.173 | <.001 |

Supplementary Table S4 Current status of medical staff allocation for cancer prevention and treatment at different levels

|  | All hospitals （M±SD） | All hospitals （P50（P25,75）） | Tertiary hospitals（P50（P25,75） | Secondary hospitals（P50（P25,75） | Z | P |
| --- | --- | --- | --- | --- | --- | --- |
| physicians | 8.03±7.47 | 7（5，9） | 8（6，11） | 5（3，6.25） | -5.678 | <0.001 |
| Chief physician | 0.98±4.49 | 0（0，1） | 1（0，1） | 0（0，1） | -2.525 | 0.012 |
| Deputy chief physician | 1.98±3.89 | 1（1,2） | 2（1，3） | 1（0，2） | -4.512 | <0.001 |
| Resident physician | 3.75±5.48 | 3（2,5） | 3（2，5） | 2（1，3） | -5.229 | <0.001 |
| Doctors with doctoral degrees | 0.23±0.97 | 0（0，0） | 0（0，0） | 0（0，0） | -1.965 | 0.049 |
| Doctor with a Master's Degree | 1.32±2.84 | 0（0，1.5） | 1（0，2） | 0（0，0） | -5.277 | <0.001 |
| nurse practitioner | 14.9±11.81 | 12（9，18） | 14（10，20） | 10.5（6，14） | -4.356 | <0.001 |
| Radiotherapy Physicist | 0.74±1.27 | 0（0，1） | 0（0，2） | 0（0，0） | -4.886 | <0.001 |
| Radiotherapy technician | 0.74±1.25 | 0.（0，1） | 0（0，2） | 0（0，0） | -4.440 | <0.001 |
